# The Second International Meeting on Microarray Data Standards, Annotations, Ontologies and Databases

**DOI:** 10.1002/1097-0061(20000930)17:3<238::AID-YEA35>3.0.CO;2-X

**Published:** 2000

**Authors:** Andy Hayes

**Affiliations:** School of Biological Sciences University of Manchester Stopford Building,Room 2.205 Oxford Road Manchester M13 9PT UK


**Yeast**
*Yeast* 2000; **17**: 238–240.

**Meeting Review**

### The Second International Meeting on Microarray Data Standards, Annotations, Ontologies and Databases

25–27 May 2000, Deutsches Krebsforschungszentrum and European Molecular Biology Laboratory, Heidelberg, Germany

Andy Hayes*
*School of Biological Sciences, University of Manchester, Oxford Road, Manchester M13 9PT, UK*

**Correspondence to:
Andy Hayes, School of Biological Sciences, University of Manchester, Stopford Building, Room 2.205, Oxford Road, Manchester M13 9PT, UK.
E-mail: andy.hayes@man.ac.uk*

### Background

In November 1999, the first international meeting on Microarray Gene Expression Databases (MGED) was held at the European Bioinformatics Institute in Hinxton. The goal of this meeting was to create a framework for developing standards for storing and communicating microarray-based gene expression data. To achieve this goal, the participants of the meeting established five working groups, each of which would be charged with addressing a particular aspect of the task in hand. Also at this meeting, a set of 'general recommendations' was drafted [1] and a follow-up meeting scheduled for the following year.

The second MGED meeting [2] took place on 25–27 May 2000 in Heidelberg, Germany, and was hosted at two sites: Deutsches Krebsforschungszentrum (DKFZ) on Thursday and Friday, then at the European Molecular Biology Laboratory (EMBL) on the Saturday. Martin Vingron (DKFZ) chaired the organizing committee, and Alvis Brazma (EBI) chaired the committee that put together the programme. Whilst the number of attendees at the meeting was limited to 250, it brought together delegates from the largest microarray groups throughout the world, both from academia and the industrial sector.

### Publications in the genomic era

Michael Eisen (Stanford) opened the proceedings with a keynote address in which he presented his visions of what scientific publications of the future may look like. He foresaw a move away from 'traditional' printed journals towards an electronic infrastructure without the space constraints imposed by conventional media. Eisen outlined several potential benefits of such a freely accessible, electronic scientific 'press', whilst also addressing the concerns about how the 'peer-review' process employed by print journals could be maintained.

### News from the working groups

In the next session of talks, the chairs from each of the working groups gave a summary of the progress, and indeed goals, of each group. The first presentation, by Alvis Brazma (EBI), was the report from the 'Microarray Data Representation and Annotation Standards' working group. Here, Alvis presented the history, state of the art and goals of this meeting and ultimately a draft proposal [3] for how array-based gene expression data should be annotated. The crux of the proposal was to define the minimum information about a

Copyright © 2000 John Wiley & Sons, Ltd.

published microarray experiment to ensure its interpretability and reproducibility.

In the second talk, Paul Spellman (Berkeley) reported from the 'Microarray Data Representation in XML' working group on their intentions to converge the different formats of XML into a single usable form. Mike Bittner (NHGRI) presented the third report, from the 'Ontologies for Microarray Experiment Description' working group. Here, he highlighted the need for a detailed and comprehensive ontology. Ideally, researchers would simply like to 'plug in' to existing ontologies. The problem is that very few ontologies actually exist and, where they do, they are generally species-specific and have not been developed for cross-organism comparisons. In the fourth talk, Frank Holstege (UMC, Utrecht) reported for the 'Normalization, Quality Control and Cross-platform Comparison' working group. Three normalization methods are currently in general use: housekeeping genes, all genes, and externally spiked control RNAs. Frank proposed that each of these methods has its advantages and disadvantages but that none was ideal. To illustrate this point, he used an experiment comparing the transcriptome of yeast cells growing in mid-exponential phase with those in stationary phase. When the data from this experiment was analysed using the different normalization strategies, each revealed a completely different profile of up- and downregulated transcripts. The suggestion from this group was that it would be inappropriate (indeed impossible) to force people to use certain normalization methods. Much better to submit raw data to the database and let the user decide on the most applicable normalization technique. In the final talk of the session, Martin Vingron (DKFZ), chair of the 'Data Queries and Mining' working group, described the types of questions users might wish to ask of microarray data. The individual groups convened at various times throughout the course of the meeting and then reported their conclusions at the end of the meeting.

### YAMAD (yet another microarray database)

The next session of talks was introduced by Terry Gaasterland (Rockefeller University) as a 'marathon of databases'. A marathon it was too! Seventeen different groups, from both academia and industry and from Europe, the USA and Japan, reported on their efforts to establish repositories and analysis tools for microarray data. The fact that such enormous effort is being channelled into these developments is perhaps testimony to the scale and complexity of the task and, indeed, the urgency for its implementation. Space constraints in this report preclude even listing the titles (and the obligatory acronyms) of the databases. During the session one of the speakers suggested that YAMAD (for yet another microarray database) might have been an appropriate name for the session. Perhaps of interest to the community at large, though, were those that aim to provide *public*, open-source facilities. These were presented by groups from both sides of the Atlantic. Alex Lash presented the NCBI's Gene Expression Omnibus (GEO) and Harry Mangalam described the GeneX database from NCGR. Alan Robinson (EBI, Hinxton) described the current status of the European effort, ArrayExpress, and David Hancock (University of Manchester) reported its relational implementation in Manchester (MaxD [4]). Each of these approaches has common elements and strives to provide a public archive for data from multiple platforms – to provide for expression data that which EMBL/GenBank/DDBJ does for DNA sequencing.

### The scale of things

The amount of data being generated from microarray experiments is potentially enormous: from a 'trickle' in the latter half of the last decade we are now seeing a 'flood' developing, no doubt, into a 'raging torrent' within the very near future. The numbers are quite staggering. The NCI's database currently holds some 7.2 million cDNA expression points. In the Stanford database alone, Mike Cherry (SGR, Stanford) anticipated a billion rows of data and 0.75 terabytes of images per year by the end of 2000. Alan Robinson envisaged storing and analysing petabytes of data in the near future and mentioned talks that the EBI were having with CERN on how they manage such vast amounts of data.

### Data normalization and quality control

Throughout the next sessions, several speakers drew attention to recent advancements in both the technology and the approaches to microarray data

Copyright © 2000 John Wiley & Sons, Ltd. 

analyses. Wilhelm Ansorge (EMBL, Heidelberg) spoke about his group's development of 're-usable' microarrays, and Jack Gerson (Gene Logic Inc.) explained the finer subtleties of optimizing for scanner saturation to improve data quality. Rick Johnston (Incyte Genomics Inc.) gave an impassioned talk comparing, 'from 50 000 feet', the performance of printed cDNA arrays with oligonucleotide 'chips'. The people at Incyte looked at some 5000 genes and, using novel performance metrics, concluded that the cDNA approach performed better in terms of precision and linearity. Roger Bumgarner (University of Washington) stressed the importance of appropriate controls and replicates. He defined their minimum unit of measure for an array experiment as containing replicates within an array, replicate arrays and also the 'flip' data (a further set of experiments where the different dyes used to label the test and reference sample are interchanged). Michael Eisen's proposal to post a dataset on the Web was very warmly received. His idea was that groups could store and analyse the (same) data using their own preferred approaches, extract any biological insight and then compare these results at a follow-up meeting.

### Related initiatives

In the following session, Scott Markel (NetGenics Inc.) reported on the activities of the Life Sciences Research branch of the Object Management Group (OMG). The OMG creates and popularizes object-orientated standards and is the world's largest software consortium, comprising over 850 member companies. This group issued a request for proposal (RFP-7) on Gene Expression on 10 March 2000, with the aim of defining interfaces, structures and models for microarray data. The outcome of this process, the final adoption vote, is due in May 2001. Gwyn Morgan (SB Pharmaceuticals, King of Prussia) then spoke about the application of genomics in risk assessment proposed by the Health and Environmental Sciences Institute.

### To the future...

Following Anne-Marie Poustka's (DKFZ, Heidelberg) keynote address describing work with the RZPD (Berlin) mouse arrays, the working groups presented their conclusions. Each speaker undertook to implement the recommendations of the various groups and outlined time-scales and work plans for these tasks. To facilitate this, a 22-person steering committee was established, comprising representatives from each group along with other key players from the microarray community. The 'Annotations' group will draft a minimum specification document and distribute it to the mailing list for approval. The 'XML' group undertook to submit their proposal to OMG by 28 July and the 'Ontologies' group to compile a draft within 1 month. The 'Normalization' workgroup considered the most appropriate strategy to be one of 'power to the people' or 'let the user decide'. The inference here was that users should submit image files along with raw data (and error estimates for each intensity). A set of standard normalization protocols would be then available for users to select the most appropriate one for their application. Also, a set of standard DNA controls should be defined (and ideally made available) for individual groups to incorporate into their own microarrays. At present we are perhaps only scratching the surface of the wealth of information that microarrays can reveal. Thanks to the teams from Hinxton and Heidelberg, we should see at the next MGED meeting (tentatively scheduled for the Spring 2001 at Stanford), the tools in place (and publicly available) with which biologists can begin to realise the full potential of this technology.

### References

1. **http://www.ebi.ac.uk/microarray/MGED/MGED14111999/index.html**
2. **http://www.ebi.ac.uk/microarray/MGED/MGED25052000/index.html**
3. **http://www.ebi.ac.uk/microarray/MGED/Annotations-wg/mged25052000-annot.html**
4. **http://www.bioinf.man.ac.uk/microarray/resources.html**

Copyright © 2000 John Wiley & Sons, Ltd. 

